# Nasal type of extranodal NK/T-cell lymphoma presenting as an atypical skin defect: A case report

**DOI:** 10.1097/MD.0000000000049584

**Published:** 2026-07-03

**Authors:** Yoo Jin Kim, In-Ki Park, Seung-Hyun Nam, Kyu Yeon Won, Jae-Ho Shin, Sang Woong Moon, Junkyu Chung

**Affiliations:** a Department of Ophthalmology, Kyung Hee University Hospital at Gangdong, Kyung Hee University, College of Medicine, Seoul, Korea; b Department of Ophthalmology, Kyung Hee University Medical Center, Kyung Hee University, College of Medicine, Seoul, Korea; c Department of Hemato-Oncology, Kyung Hee University Hospital at Gangdong, Kyung Hee University, College of Medicine, Seoul, Korea; d Department of Pathology, Kyung Hee University Hospital at Gangdong, Kyung Hee University, College of Medicine, Seoul, Korea.

**Keywords:** case report, Epstein–Barr virus-negative, extranodal NK/T-cell lymphoma, eyelid ulcer, nasal type

## Abstract

**Rationale:**

Nasal-type extranodal natural killer/T-cell lymphoma (ENKTL) is a rare malignancy primarily affecting the upper aerodigestive tract. It is strongly associated with Epstein–Barr virus (EBV) infection; thus, EBV-negative cases are extremely rare, and their clinicopathological features and treatment responses are not well defined.

**Patient concerns:**

A 75-year-old male presented with a persistent skin defect in the medial canthal area of the left eye. He had been treated with antibiotics for presumed cellulitis for 2 months, but the symptoms persisted, and the skin defect worsened.

**Diagnoses:**

Surgical debridement revealed an irregularly shaped white mass extending into the paranasal sinus. Incisional biopsy revealed atypical lymphocytic infiltration favoring ENKTL. EBV in situ hybridization was negative, supporting the diagnosis of EBV-negative ENKTL. Staging examinations, including whole-body computed tomography and positron emission tomography, revealed localized disease involving only the orbit and paranasal sinuses.

**Interventions:**

The patient was treated with etoposide, ifosfamide, cisplatin, and dexamethasone chemotherapy. After the first cycle, the patient developed severe neutropenia, and the chemotherapy dose was reduced by 40% for the second cycle. After 2 cycles of etoposide, ifosfamide, cisplatin, and dexamethasone chemotherapy, consolidation radiation therapy was recommended.

**Outcomes:**

After 2 cycles of chemotherapy, positron emission tomography showed metabolic complete remission. At 3 months after the second cycle of chemotherapy, the skin defect had completely healed, and follow-up computed tomography showed disease remission.

**Lessons:**

Cutaneous manifestations of ENKTL can mimic benign inflammatory conditions such as cellulitis, leading to delayed diagnosis. Clinicians should maintain a high index of suspicion and perform early tissue biopsy for atypical ulcerative lesions in the periorbital and nasal areas, even in the absence of typical EBV positivity.

## 1. Introduction

Nasal-type extranodal natural killer/T-cell lymphoma (ENKTL) is a rare extranodal non-Hodgkin lymphoma recognized in the World Health Organization lymphoma classification systems.^[1,2]^ Bakos et al^[3]^ reported a higher prevalence in Asia and Latin America, where ENKTL accounts for approximately 10% of all non-Hodgkin lymphoma cases, compared with <1% in Europe and North America. Because ENKTL is highly aggressive, delayed or missed early diagnosis is closely linked to a significantly worse prognosis.^[4]^ Therefore, early diagnosis and timely and appropriate treatment are essential for a good prognosis. Most ENKTLs are strongly associated with Epstein–Barr virus (EBV) infection, and clonal EBV episomes are typically found within tumor cells, suggesting a potential role in tumor pathogenesis.^[5]^ However, EBV-negative ENKTLs are so rare that whether they should be grouped with EBV-positive ENKTLs is controversial. Furthermore, the known clinicopathological characteristics are limited.

Recent studies across immunology and cancer biology have emphasized that disease behavior and treatment response are influenced not only by histopathologic classification but also by immune-checkpoint pathways and cytokine-mediated inflammatory signaling.^[6–8]^ Intracellular signaling networks and protein conformation-based target characterization have also been investigated as mechanisms relevant to disease biology and therapeutic development.^[9,10]^ In oncology, cancer stemness and epigenetic or metabolic regulators such as sirtuins have been implicated in tumor progression, recurrence, treatment resistance, and therapeutic vulnerability.^[11–13]^ In hematologic malignancies, target-antigen selection is also central to emerging cellular therapies such as chimeric antigen receptor T-cell therapy.^[14]^

Herein, we report a case of nasal-type EBV-negative ENKTL that showed remission after chemotherapy.

Written informed consent was obtained from the patient for the publication of this case report. Ethical committee approval was not required as this was a retrospective description of a single case and did not involve prospective research procedures.

## 2. Case presentation

**Figure 1.**
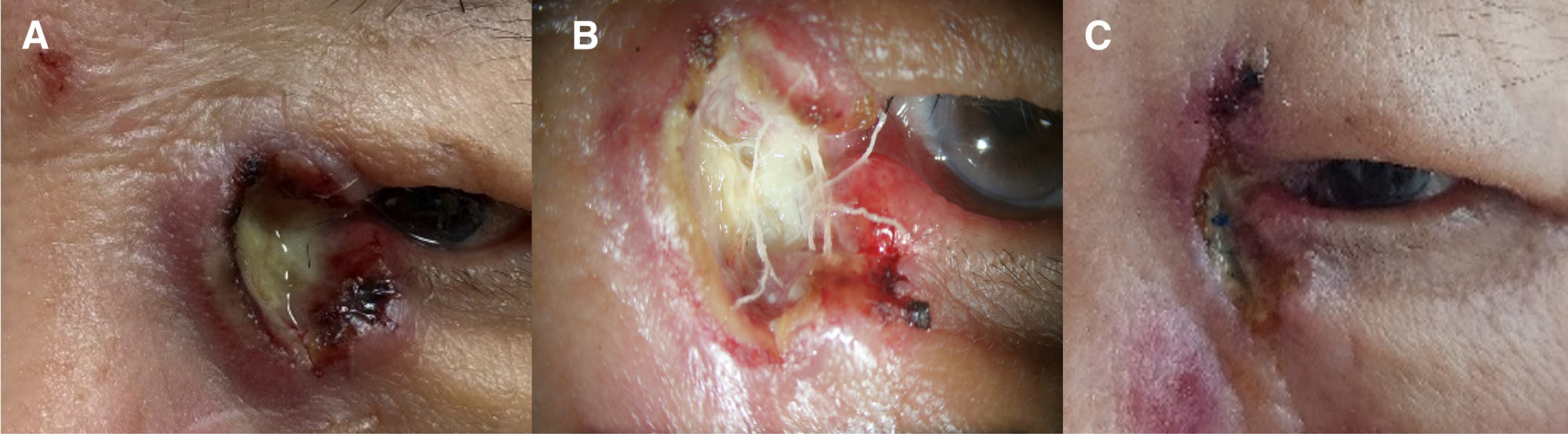
Clinical photographs of the left medial canthal lesion. (A) A large ulcerative skin defect was observed in the medial canthal area of the left eye at initial presentation. (B) After surgical debridement, a palpable, irregularly shaped white mass was identified beneath the necrotic tissue. (C) The remaining skin defect was approximated using 5-0 Prolene after incisional biopsy.

**Figure 2.**
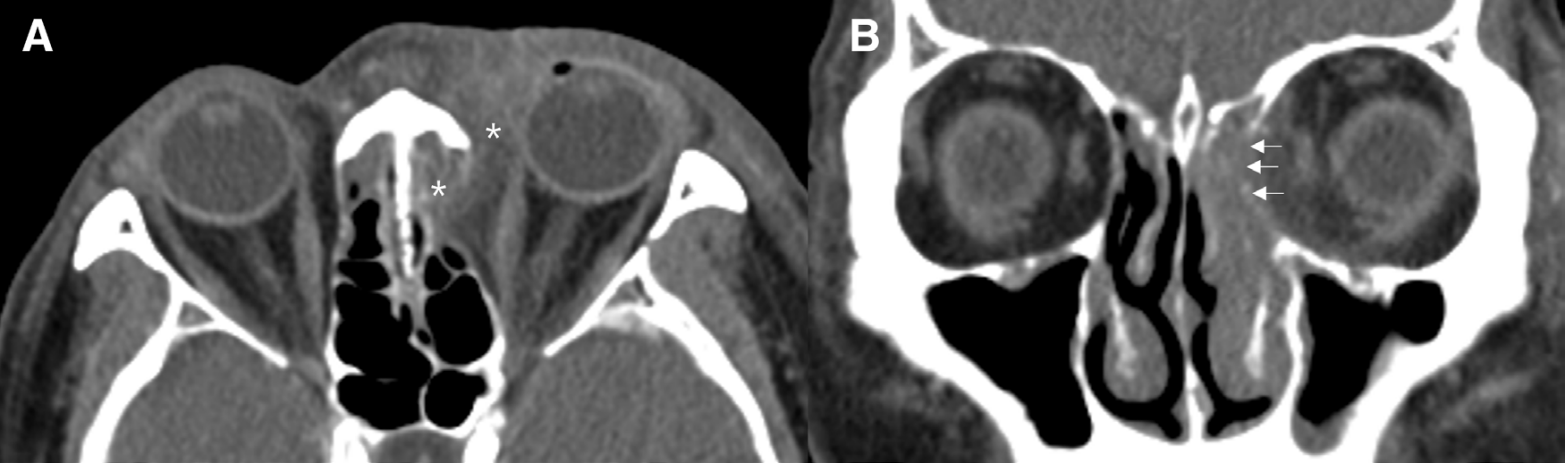
Facial computed tomography images obtained on the day of surgery. (A) Axial view showing a soft tissue mass invading the left ethmoid sinus and medial orbit (*). (B) Coronal view showing bone destruction of the left medial orbital wall (arrows).

**Figure 3.**
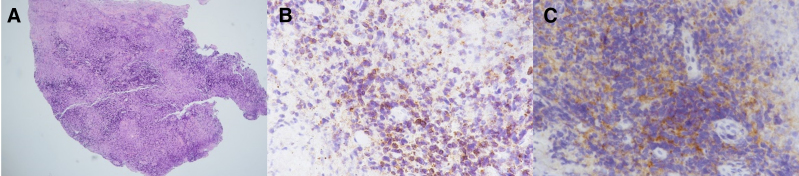
Histopathologic and immunohistochemical findings of the left medial canthal mass. (A) Hematoxylin and eosin staining showed atypical lymphocytic infiltration in the biopsied eyelid mass. Immunohistochemical staining demonstrated CD3 positivity (B) and CD56 positivity (C), supporting the diagnosis of extranodal NK/T-cell lymphoma. CD3 = cluster of differentiation 3, CD56 = cluster of differentiation 56, NK = natural killer.

**Figure 4.**
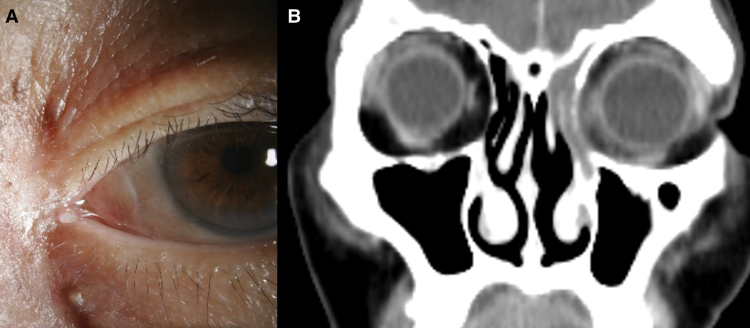
Clinical and radiologic findings 3 months after the second cycle of chemotherapy. (A) Clinical photograph showing complete healing of the left medial canthal skin defect. (B) Follow-up facial computed tomography showing remission of the previously noted soft tissue mass involving the left ethmoid sinus and medial orbit.

A 75-year-old male presented with an approximately 3.5 × 2.5-cm skin defect in the medial canthal area of the left eye (Fig. 1A). He had a medical history of diabetes mellitus. He had no relevant family history, was a never-smoker, and did not consume alcohol. No relevant psychosocial history was identified. At initial presentation, visual acuity was 20/25 in the right eye and 20/63 in the left eye, with no improvement on correction. Intraocular pressure was 16 mm Hg in the right eye and 19 mm Hg in the left eye. Slit-lamp examination was unremarkable except for age-related cataracts. Two months prior, the patient had been diagnosed with cellulitis and was treated at another hospital for redness and swelling in the medial canthal area of the left eye. However, the symptoms persisted, and the patient continued to pick at the irritated area, causing skin damage, leading to the visit. The treatment plan was to administer intravenous and oral antibiotics for 1 week, followed by direct closure of the defect under local anesthesia. On the day of surgery, necrotic tissue was debrided for direct suturing, revealing a palpable, irregularly shaped white mass extending into the paranasal sinus (Fig. 1B). An incisional biopsy of the tumor was performed, and the defect was closed as much as possible using 5-0 Prolene to complete the surgery (Fig. 1C). Facial computed tomography (CT) with contrast medium performed on the day of surgery revealed a soft tissue mass suspicious for malignancy, invading the left ethmoid sinus and medial orbit, with bone destruction of the left medial orbital wall (Fig. 2). Six days after surgery, the biopsy of the eyelid mass revealed atypical lymphocytic infiltration favoring ENKTL (Fig. 3). Immunohistochemical staining showed positivity for cluster of differentiation 45RB/leukocyte common antigen, cluster of differentiation 56, cluster of differentiation 3, granzyme B, and T-cell intracellular antigen-1, whereas cluster of differentiation 20, cytokeratin AE1/AE3, and synaptophysin were negative. The Ki-67 labeling index was approximately 30%. Molecular pathology testing using EBV in situ hybridization was performed on an adequate specimen and showed negative results, supporting the diagnosis of EBV-negative ENKTL. The patient was subsequently transferred to the Department of Hematology and Oncology. Subsequent whole-body CT scan and positron emission tomography showed no evidence of disease involvement outside the orbit and paranasal sinuses. The patient was administered chemotherapy with etoposide, ifosfamide, cisplatin, and dexamethasone (VIPD). After the first cycle, the patient developed severe neutropenia, and the chemotherapy dose was reduced by 40% for the second cycle. After 2 cycles of VIPD chemotherapy, positron emission tomography showed metabolic complete remission, and consolidation radiation therapy was recommended. At 3 months after the second cycle of chemotherapy, the skin defect had healed, and follow-up CT showed disease remission (Fig. 4).

## 3. Discussion

Nasal-type ENKTL is a rare disease that mainly affects the upper respiratory and digestive systems. The clinical features vary depending on the affected area, making accurate early-stage diagnosis difficult.^[15]^ EBV infection is associated with the cytotoxic and tissue-destructive characteristics of ENKTL, and clonal EBV is typically found within tumor cells in most EBV-positive cases.^[5,16]^ However, EBV-negative ENKTL is even rarer and is primarily reported in small case series or case reports from Asia. Two of the 3 previous studies comparing prognosis and clinicopathological differences between EBV-negative and EBV-positive cases reported no significant differences. However, a larger study reported less aggressive necrosis in EBV-negative cases, which was attributed to EBV-induced upregulation of cytokines such as interferon-gamma-inducible protein.^[17]^

Treatment for localized EBV-negative ENKTL should be based on the treatment for localized ENKTL. However, the treatment of localized ENKTL remains limited owing to the lack of randomized controlled trials and reliance on the results of phase II trials and retrospective analyses. Therefore, a standard treatment strategy has not been firmly established, although combined treatment with chemotherapy and radiotherapy is recommended. Chemotherapy should be selected by the physician according to the patient’s condition and risk level.^[18]^ The prognosis of ENKTL is largely determined by disease stage. The 5-year survival rate is 55% to 90% for low-, intermediate-, and high-risk early-stage disease, and <40% for high-risk, advanced-stage disease. Thus, early suspicion is critical; however, cutaneous manifestations, as in this case, may mimic cellulitis and abscesses and delay recognition of ENKTL, as in the present case.^[19]^ As diagnosis is made through tissue biopsy and immunohistochemistry, this disease should be suspected, and tissue biopsy should be performed in patients presenting with a large, ulcerative lesion of unknown etiology around the nose. In the present case, the diagnosis was supported by atypical lymphocytic infiltration and an immunophenotype consistent with ENKTL, including positivity for cluster of differentiation 45RB/leukocyte common antigen, cluster of differentiation 56, cluster of differentiation 3, granzyme B, and T-cell intracellular antigen-1, and negativity for cluster of differentiation 20, cytokeratin AE1/AE3, and synaptophysin. EBV in situ hybridization was negative, supporting the diagnosis of EBV-negative disease.

Beyond the diagnostic aspect, recent literature highlights several biological and therapeutic directions that may be relevant to future studies of rare lymphoid malignancies, including EBV-negative ENKTL. Immune-checkpoint pathways such as programmed death-1/programmed death-ligand 1 and cytokine-driven inflammatory networks, including interleukin-11 and NOD-like receptor family pyrin domain-containing 3 inflammasome activation, may influence immune dysregulation and host–tumor interactions.^[6–8]^ Intracellular signaling regulators such as Src homology 2 domain-containing phosphatase-1/cluster of differentiation 40 and protein conformation-based target characterization further illustrate mechanism-based therapeutic exploration across disease models.^[9,10]^ In parallel, cancer stemness and epigenetic or metabolic regulators, including sirtuins, have been implicated in tumor progression, recurrence, and treatment resistance across different cancers.^[11–13]^ Furthermore, the development of target-antigen-based cellular therapy in hematologic malignancies underscores the growing importance of mechanism-based approaches in rare or refractory malignancies.^[14]^

Although concurrent radiotherapy and chemotherapy are the most commonly recommended treatments for localized ENKTL, chemotherapy alone was initiated at our hospital. After the first VIPD cycle, the patient experienced severe neutropenia, requiring a reduced dose for the second cycle. The patient achieved metabolic complete remission after 2 cycles of VIPD chemotherapy, and the skin defect improved with granulation.

The strength of this report is that it documents the diagnostic process and initial treatment response in a rare case of EBV-negative ENKTL, providing a reference for similar clinical scenarios. However, this study is limited by its nature as a retrospective single case report, which restricts the generalizability of the findings.

In conclusion, this case highlights that nasal-type ENKTL can initially mimic benign inflammatory conditions such as cellulitis or chronic skin ulceration. Early biopsy should be considered for atypical, persistent ulcerative lesions in the periorbital and nasal regions. A comprehensive histopathologic, immunohistochemical, and molecular evaluation is essential for accurate diagnosis. In localized disease, multidisciplinary treatment, including chemotherapy and planned consolidation radiotherapy, may provide clinical benefit, although the optimal treatment strategy for EBV-negative cases remains to be further defined.

## Author contributions

**Writing – original draft:** Yoo Jin Kim, In-Ki Park, Seung-Hyun Nam, Kyu Yeon Won, Jae-Ho Shin, Sang Woong Moon, Junkyu Chung.

**Writing – review & editing:** Yoo Jin Kim, In-Ki Park, Seung-Hyun Nam, Kyu Yeon Won, Jae-Ho Shin, Sang Woong Moon, Junkyu Chung.
